# Pharmacogenetics and phenoconversion: the influence on side effects experienced by psychiatric patients

**DOI:** 10.3389/fgene.2023.1249164

**Published:** 2023-08-25

**Authors:** Manon G. den Uil, Hannelotte W. Hut, Kay R. Wagelaar, Heshu Abdullah-Koolmees, Wiepke Cahn, Ingeborg Wilting, Vera H. M. Deneer

**Affiliations:** ^1^ Division Laboratories, Pharmacy and Biomedical Genetics, Clinical Pharmacy, University Medical Centre Utrecht, Utrecht, Netherlands; ^2^ Department of Clinical Pharmacy, Medisch Spectrum Twente, Enschede, Netherlands; ^3^ Pharmacy and Clinical Pharmacology, Amsterdam University Medical Center, Vrije Universiteit Amsterdam, Amsterdam, Netherlands; ^4^ Division of Pharmacoepidemiology and Clinical Pharmacology, Utrecht Institute for Pharmaceutical Sciences, Utrecht University, Utrecht, Netherlands; ^5^ Department of Psychiatry, Rudolf Magnus Institute of Neuroscience, University Medical Center Utrecht, Utrecht, Netherlands

**Keywords:** pharmacogenetics, phenoconversion, side effects, psychiatric drugs, CYP2C19, CYP2D6

## Abstract

**Introduction:** Preventing side effects is important to ensure optimal psychopharmacotherapy and therapeutic adherence among psychiatric patients. Obtaining the pharmacogenetic profile of *CYP2C19* and *CYP2D6* can play an important role in this. When the genotype-predicted phenotype shifts because of the use of co-medication, this is called phenoconversion. The aim was to study the influence of the pharmacogenetic (PGx) profile and phenoconversion on side effects experienced by psychiatric patients.

**Methods:** A retrospective cohort study was performed using data from 117 patients from a psychiatric outpatient clinic. Patients were genotyped with a psychiatric PGx panel and side effects were evaluated using the *Udvalg for Kliniske Undersølgelser* side effects rating scale (UKU).

**Results:** Of all patients, 10.3% and 9.4% underwent phenoconversion (any shift in predicted phenotype) for *CYP2C19* and *CYP2D6* respectively. No significant associations were found between the phenotype and UKU-score. 75% of the patients with an Intermediate metabolizer (IM) or Poor metabolizer (PM) phenoconverted phenotype of *CYP2C19* experienced nausea and vomiting compared to 9.1% of the Normal metabolizer (NM) and Ultrarapid metabolizer (UM) patients (*p* = 0.033). 64% of the patients with an IM or PM phenoconverted phenotype of *CYP2D6* experienced the side effect depression compared to 30.4% NMs and UMs (*p* = 0.020). *CYP2D6* IM and PM patients had a higher concentration-dose ratio than NM patients (*p* < 0.05).

**Discussion:** This study underlines the importance to consider phenoconversion when looking at a patient’s genotype. This is important for a better prediction of the phenotype and preventing possible side effects under a specific psychopharmacotherapy.

## 1 Introduction

A high prevalence of polypharmacy is seen among psychiatric patients (Hefner et al., 2020). Polypharmacy, the use of five or more drugs, is often associated with drug-drug interactions and the risk of side effects. Preventing these side effects is important to ensure optimal psychopharmacotherapy and therapeutic adherence (Bousman et al., 2021).

The use of pharmacogenetics (PGx) contributes to individual patient treatment and can play an important role in preventing side effects (Sharp et al., 2019). PGx can distinct the different genetic variants of genes encoding for cytochrome P450 enzymes (CYP) such as CYP2C19 and CYP2D6 with a different metabolic capacity (KNMP, 2022a; KNMP, 2022b). A patient’s genotype can be translated into the following predicted phenotypes: normal metabolizer (NM), intermediate metabolizer (IM), poor metabolizer (PM) or ultrarapid metabolizer (UM) (Brouwer et al., 2022). Different consortia such as the Dutch Pharmacogenetics Working Group (DPWG) or the Clinical Pharmacogenetics Implementation Consortium (CPIC) have written guidelines regarding dose and pharmacotherapeutic recommendation for each genotype with an actionable drug-gene interaction (DGI) (Abdullah-Koolmees et al., 2021).

CYP2C19 and CYP2D6 are responsible for the metabolism of many psychiatric drugs (Abdullah-Koolmees et al., 2021; Brouwer et al., 2022). In addition, these are also highly polymorphic enzymes and therefore pharmacogenetic advise on these DGIs are widely available (Bousman and Dunlop, 2018; Hahn and Roll, 2021). Furthermore, PGx can help improve and optimize pharmacotherapy for individual patients using psychiatric drugs. Other CYP-enzymes such as CYP1A2, CYP2C9 and CYP3A4 can also play a role in the metabolism of psychiatric drugs. However, the impact of their genotypes on the pharmacokinetics of commonly used psychiatric drugs is less distinct as compared to the effect of *CYP2C19* and *CYP2D6* genotypes. Previous studies have reported that patients with a predicted phenotype of PM for either *CYP2C19* or *CYP2D6* have a higher risk of side effects (Chou et al., 2000; Kobylecki et al., 2009; Mrazek et al., 2011). However, in other studies no association between the genotype and the development of side effects has been observed (Peters et al., 2008; Hodgson et al., 2015).

Other non-genetic factors, such as co-medication, also influence the patient’s phenotype, i.e., a patient’s metabolic capacity (Klomp et al., 2020). This can have a significant impact, especially on patients with polypharmacy. This phenomenon, where the predicted metabolic capacity shifts because of the use of co-medication or other non-genetic factors, is called phenoconversion (Hahn and Roll, 2021). In this study, phenoconversion by co-medication will be taken into account.

Research shows that CYP-inhibition or CYP-induction by co-medication often has the greatest influence on NMs and IMs, causing a change in the drug exposure (AUC) (Bahar et al., 2017). For instance, it has been shown that patients using (es)citalopram are more prone to a dose reduction or switching to another antidepressant when there is a drug-drug-interaction combined with a DGI with *CYP2C19* (MuhA et al., 2020). However, the relationship between side effects, pharmacogenetic profile and phenoconversion remains to be studied. The aim was to study side effects experienced by psychiatric patients and to identify risk factors including but, not limited to, pharmacogenetic profile of *CYP2C19* and *CYP2D6* and phenoconversion.

## 2 Materials and methods

### 2.1 Study design and population

A retrospective cohort study was performed using the data of the “Body and Life” project (Dutch: *Lijf en Leven*), for which a non-WMO acknowledgment from the Medical Research Ethics Committee Utrecht has been authorized (number 19-447/C). All patients gave written informed consent. For this study, psychiatric patients were enrolled from the outpatient clinic also named “Body and Life” (Dutch: *Lijf en Leven*, LL-clinic) of the department of psychiatry from the University Medical Center Utrecht (UMC Utrecht) in the Netherlands (UMC Utrecht, 2022a). Patients were excluded if no genotyping was performed. Patients enrolled between February 2018 and March 2022 were included in the analysis. Data was extracted from the electronic health record.

For the comparison of the distribution of the genotypes, control populations were used. For the control population of *CYP2C19*, a group of 820 coronary artery disease (CAD) patients who underwent elective coronary stenting was used. For the control population of *CYP2D6*, a group of 134 healthy controls recruited from hospital personnel was used. These patients have been genotyped as part of other studies, which have been approved by Medical Research Ethics Committee of St. Antonius Hospital Nieuwegein, the Netherlands.

### 2.2 Drug classification and phenoconversion

The current drug use was documented. Within the drugs used, CYP-substrates as well as CYP-modulators for CYP2C19 and CYP2D6 were identified. CYP-substrate users were defined as a patient who uses a CYP2C19- or CYP2D6 substrate which has a psychiatric indication and where a therapeutic recommendation is given for the DGI by the DPWG (Abdullah-Koolmees et al., 2021; KNMP, 2022c). If no advice was given for a substrate, a literature search was conducted to see if the DGI was of clinical relevance. This was only the case for diazepam, which is considered a CYP2C19-substrate with a pharmacogenetic interaction. (Qin et al., 1999; Skryabin et al., 2020; KNMP, 2022d).

A CYP-modulator was defined as a drug that has a moderate or strong inhibitory or inducing effect on CYP2C19 and/or CYP2D6 (Cicali et al., 2021). There are no known inducers for CYP2D6 (Just et al., 2021). Relevant inhibitors and inducers can be found in Supplementary Material S1. In this study, phenoconversion was defined as the shift of a patient’s phenotype based on the use of co-medication consisting of CYP-modulators. The genotype-predicted phenotype was adjusted to a phenoconverted phenotype (*P-CYP2C19* and *P-CYP2D6*) according to Table 1 (Hahn and Roll, 2021; Just et al., 2021). Phenotypes were classified in the next lower activity phenotype using a moderate inhibitor and an even lower activity phenotype when using a strong inhibitor. For example, a patient who is a *CYP2D6* NM but uses fluoxetine, a strong CYP2D6-inhibitor, is a *P-CYP2D6* PM. If a patient used a CYP2C19-inducer, a patient was classified into the next higher activity phenotype. Only PMs kept poor activity because increased synthesis of “loss of function proteins” does not change the drug clearance and thus the phenotype.

**TABLE 1:** Phenoconverted phenotype for CYP2D6 and CYP2C19 based on concomitant use of inhibitor/inducer. The presented phenotypes are what the genotype-predicted phenotypes will convert to when a moderate inhibitor, strong inhibitor or inducers is taken concomitantly.

Genotype-predicted phenotype	Moderate inhibitor	Strong inhibitor	Inducer (only for CYP2C19)
PM	PM	PM	PM
IM	PM	PM	NM
NM	IM	PM	UM
UM	NM	IM	UM

Abbreviations PM, poor metabolizer; IM, intermediate metabolizer; NM, normal metabolizer; UM, ultrarapid metabolizer.

### 2.3 Genotyping

DNA-diagnostics were performed at *Erasmus MC clinical laboratory* in Rotterdam, Netherlands (Erasmus, 2022). Genotypes were translated to corresponding phenotypes recognized by the DPWG (Abdullah-Koolmees et al., 2021; KNMP, 2022a). Patients were tested for *CYP1A2, CYP2C9, CYP2C19, CYP2D6* and *CYP3A4*, but only the genotypes of *CYP2C19* and *CYP2D6* were used. *CYP1A2* was not included because there were no actionable DGIs according to the DPWG. *CYP2C9* was not included because no patient used a CYP2C9-metabolized psychiatric drug with a relevant DGI and for *CYP3A4* were no PMs identified (only phenotype with actionable PGx recommendation).

### 2.4 Side effect registration

Side effects were evaluated using the *Udvalg for Kliniske Undersølgelser side effects rating scale (UKU)* for the registration of side effects of psychotropic drugs (Lingjærde et al., 1987). In the UKU, every side effect is rated in a four-point scale, and all scores were added together to get a total score. A higher score implies a higher rate of side effects or more severe side effects. An adapted version of the UKU-rating scale specifically for the LL-clinic was used, from which the extrapyramidal symptoms were excluded from the category neurologic side effects and can be found in the Supplementary Material S2. The UKU-questionnaire was conducted orally by a nurse specialist via a semi structured interview with the patient.

### 2.5 Concentration-dose ratios

Next to a blood sample for the DNA-diagnostics, there was also a sample send to the *pharmacy laboratory of UMC Utrecht* for determination of drug levels in plasma (UMC Utrecht, 2022b). This was done for all psychiatric drugs a patient used at the time of measurement. For each patient a blood sample was taken in the morning, with medication taken the night before but not in the morning. Based on the drug concentration (in μg/L) and the registered dose (in mg), the concentration-dose ratio (CD-ratio) was calculated in μg/l/mg for further analysis. Only the drugs with at least two users in the different phenotypic groups were included for further analysis.

### 2.6 Statistical analysis

Continuous variables were presented as mean and standard deviation (median and range if non-normal distributed) and categorical variables as frequency and percentage. Comparisons of normal distributed continuous variables were performed with a student’s T-test. For non-normal distributed variables, a Mann-Whitney U-test was used or a Kruskal Wallis test if there were more than two groups. For categorical variables, a chi-squared test was used. Only CYP-substrate users were included in the phenotype-specific analysis and NM was seen as the reference phenotype.

Associations between the UKU-score and patient characteristics were analyzed using binary logistic regression. Phenotypes were combined, i.e., NM with UM and IM with PM, because of the small sample size per phenotype group. Although there are known distinct PK differences between these phenotypes, it is hypothesized that the IMs and PMs will experience more side effects than the NMs and UMs when receiving standard doses. For the analysis of the UKU-score the score was divided into three categories (low, moderate and high) based on tertiles. An univariate as well as a multivariate analysis was performed, adjusting for body mass index (BMI), psychiatric diagnosis and polypharmacy.

The comparison of the prevalence of specific side effects was only done for CYP-related side effects, which were determined for a project of the *Ubiquitous Pharmacogenomics Consortium*. Five researchers assessed each side effect based on clinical studies, the Summary of Product Characteristics and expert-opinions. The outcomes of the assessments [unpublished], i.e., whether specific side effects of drugs are genotype dependent, was used. Of the UKU-questionnaire, 33 of the 39 side effects were considered CYP-related (see Table 5; Table 6 for the included side effects).

A *p*-value < 0.05 was considered statistically significant. All statistical analyses were performed using IBM SPSS Statistics (Version 26.0.0.1, IBM Corp., Armonk, NY, United States).

## 3 Results

**FIGURE 1:**
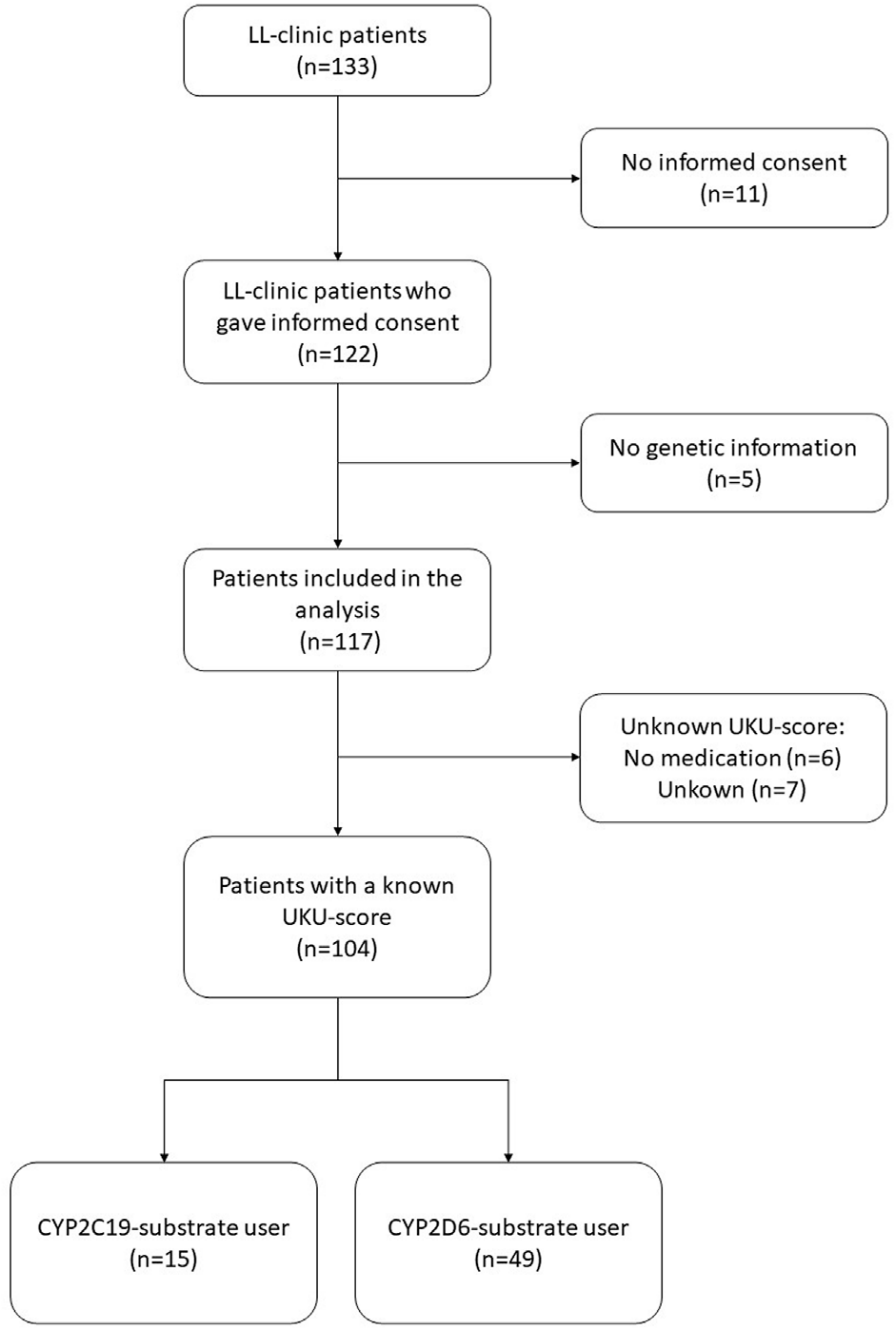
Selection of LL-clinic patients included into this study. Abbreviations *LL-patients* patients from “Body and Life” outpatient clinic; *UKU* Udvalg for Kliniske Undersølger side effects rating scale.

**FIGURE 2:**
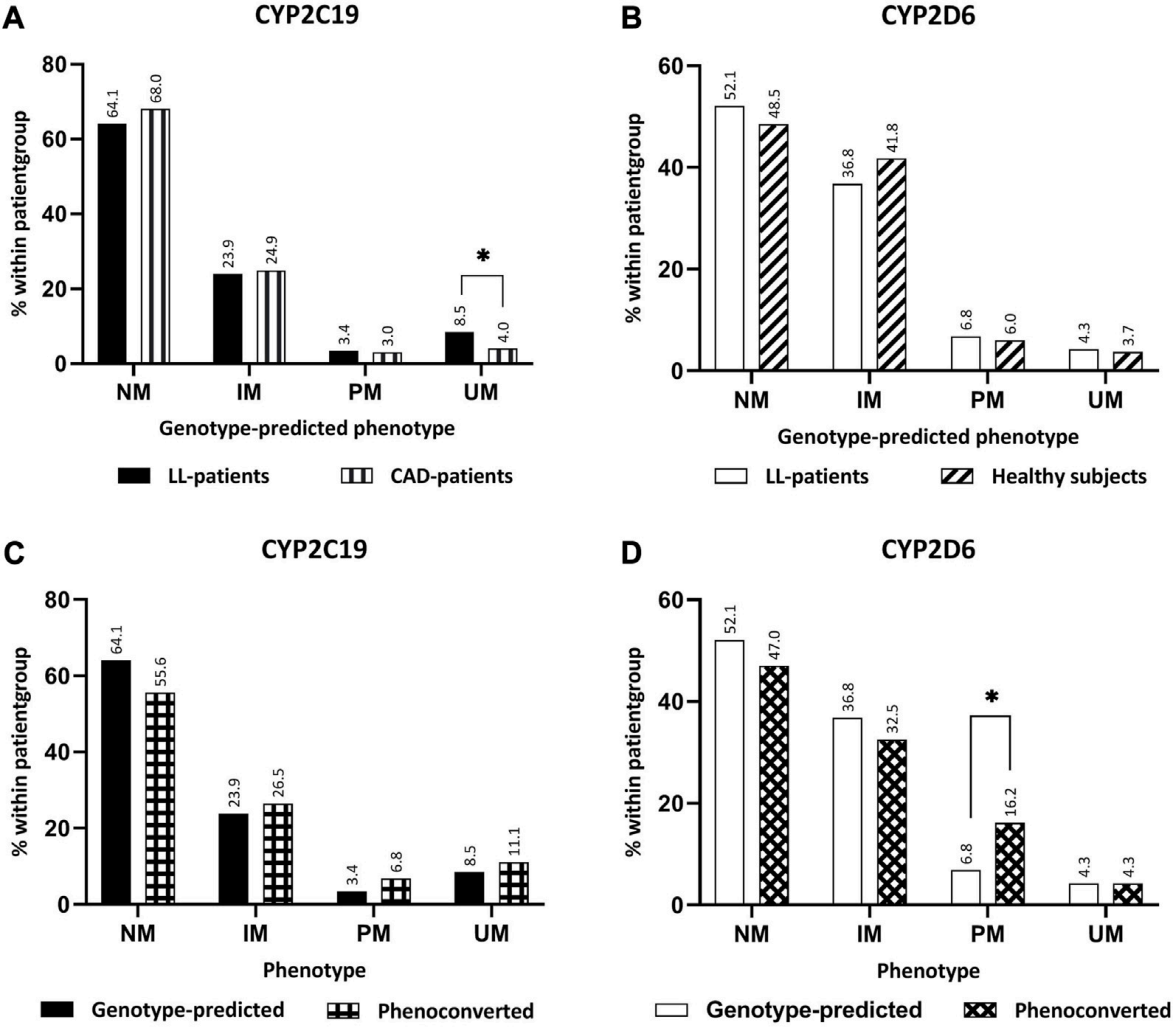
Comparison between phenotypes. **(A)** Comparison genotype-predicted phenotype of *CYP2C19* between LL-patients (*n* = 117) and CAD-patients (*n* = 820). **(B)** Comparison genotype-predicted phenotype of CYP2D6 between LL-patients (*n* = 117) and healthy subjects (*n* = 134). **(C)** Comparison of genotype-predicted phenotypes versus phenoconverted phenotypes *CYP2C19*. **(D)** Comparison of genotype-predicted phenotypes versus phenoconverted phenotypes *CYP2D6*. Significant differences (*p* < 0.05) are depicted with an *. Abbreviations *NM*, normal metabolizer; *IM*, intermediate metabolizer; *PM*, poor metabolizer; *UM*, ultrarapid metabolizer; *LL-patients* patients from “Body and Life” outpatient clinic; *CAD-patients* Coronary artery disease patients.

In total, 117 LL-patients were eligible for this study, of which 104 (88.9%) LL-patients filled in the UKU-questionnaire and could be included in the analysis (Figure 1). Of the 104 LL-patients who were included in the analysis, 15 (14,4%) LL-patients concomitantly used a CYP2C19-substrate and 49 (47,1%) a CYP2D6-substrate. Table 2 shows the baseline characteristics of the study population. Figure 2 compares the distribution of the different genotype-predicted phenotypes of *CYP2C19* and *CYP2D6*. The proportion of UMs in *CYP2C19* was significantly larger for the LL-patients (8.5%) compared to the CAD-patients (4.0%).

**TABLE 2:** Baseline characteristics of LL-patients.

	Patients
*Total*	117
*Gender (%)*	
Male	46 (39.3)
Female	71 (60.7)
*Age—in years (mean ± SD)*	42.5 ± 11.8
*Weight—in kg (mean ± SD)*	109.3 ± 28.7
*Height—in cm (mean ± SD)*	174.2 ± 10.5
*BMI—in kg/m* ^*2*^ (*mean ± SD*)	36.0 ± 9.4
*BMI classification (%)*	
Underweight (< 18,5)	1 (0.9)
Normal weight (18,5–24,9)	11 (9.4)
Overweight (25–29,9)	18 (15.4)
Obese (30–39,9)	55 (47)
Morbid obese (BMI ≥ 40)	31 (26.5)
*Main diagnosis (%)*	
Schizophrenia	43 (36.8)
Personality disorder	11 (9.4)
Bipolar mood disorder	29 (24.8)
Depressive mood disorder	13 (11.1)
Anxiety disorder	2 (1.7)
PTSD	6 (5.1)
Neurodevelopmental disorder	8 (6.8)
Somatic symptom disorder	1 (0.9)
Addiction	1 (0.9)
Eating disorder	2 (1.7)
Other	1 (0.9)
*Main diagnosis grouped (%)*	
Psychotic (schizophrenia and bipolar disorder)	72 (61.5)
Non-psychotic (depression and others)	45 (38.5)
*Amount of drugs in use* (*median* [r*ange*])	5 [0–16]
*Polypharmacy (≥5 drugs in use) (%)*	
Yes	68 (58.1)
No	49 (41.9)
*CYP2C19-substrate in use (%)*	
Yes	17 (14.5)
No	100 (85.5)
*CYP2D6-substrate in use (%)*	
Yes	52 (44.4)
No	65 (55.6)
*CYP2C19-modulator in use (%)*	
No modulator	85 (72.6)
Weak inhibitor	20 (17.1)
Moderate inhibitor	6 (5.1)
Strong inhibitor	3 (2.6)
Inducer	3 (2.6)
*Phenoconversion CYP2C19 (%)*	
Yes	12 (10.3)
No	105 (89.7)
*CYP2D6-inhibitor in use (%)*	
No inhibitor	91 (77.8)
Weak inhibitor	15 (12.8)
Moderate inhibitor	–
Strong inhibitor	11 (9.4)
*Phenoconversion CYP2D6 (%)*	
Yes	11 (9.4)
No	106 (90.6)

Abbreviations *BMI*, body mass index; *SD*, standard deviation; *PM*, poor metabolizer; *IM*, intermediate metabolizer; *NM*, normal metabolizer; *UM*, ultrarapid metabolizer.

Approximately 10% of the LL-patients underwent phenoconversion for either *CYP2C19* or *CYP2D6* (Table 2). The comparison of the distributions of CYP2D6 before and after phenoconversion, revealed a significant difference between the proportions of PMs (Figure 2). In the genotype-predicted phenotypes, 6.8% of the patients were a PM of *CYP2D6*, which was 16.2% of the *P-CYP2D6* patients (*p* < 0.05). No significant differences were found within *CYP2C19* phenotype groups.

Comparing the total UKU-score, significant differences were found for the main diagnosis when comparing a psychotic diagnosis category (median score 13) to non-psychotic category (median score 22, *p* < 0.05) (Table 3). There was also a significant difference in the total UKU-score considering polypharmacy (patients without polypharmacy had a median score of 11, patients with polypharmacy a median score of 20, *p* < 0.05). The UKU-score was higher for patients with BMI over 40, but this was not a significant difference (*p* = 0.064; median BMI < 30 is 13, median BMI 30–40 is 15 and median BMI > 40 is 23.5). No significant differences were found for age and gender.

**TABLE 3:** Comparisons of UKU-score with baseline characteristics and comparison of phenotypes CYP-substrate users and UKU-score.

Total population	Number (%)	Total score UKU (median [range])	*p*-value (*significant)
*Total with known UKU*	104 (88.9)	15.5 [0–52]	
*Age, categorized*			NS
43 or younger (ref)	52 (50.0)	16 [0–49]	
44 or older	52 (50.0)	13 [2–52]	
*Gender*			NS
Male (ref)	43 (41.3)	14 [2–52]	
Female	61 (58.7)	16 [0–51]	
*BMI, categorized*			0.064
< 30 (ref)	26 (25.2)	13 [2–51]	
30–40	51 (49.5)	15 [0–52]	NS
>40	26 (25.2)	23.5 [6–49]	0.126
*Main diagnosis*			0.002*
Psychotic (ref)	68 (65.4)	13 [0–46]	
Non-psychotic	36 (34.6)	22 [2–52]	
*Polypharmacy*			<0.001*
Yes	63 (60.5)	20 [4–52]	
No (ref)	41 (39.4)	11 [0–51]	
Only CYP-substrate users			
*P-CYP2C19*	15 (14.4)		NS
NM (ref)	9	16 [4–27]	
IM	3	28 [12–52]	0.115
PM	1	–	NS
UM	2	18 [16–20]	NS
*P-CYP2D6*	49 (47.1)		0.089
NM (ref)	21	14 [2–37]	
IM	16	19.5 [4–52]	0.145
PM	10	15 [6–46]	NS
UM	2	43.5 [36–51]	0.029*
*CYP2C19*	15 (14.4)		NS
NM (ref)	11	16 [4–27]	
IM	3	28 [12–52]	0.101
UM	1	–	N.A.
*CYP2D6*	49 (47.1)		0.203
NM (ref)	25	18 [2–37]	
IM	19	16 [4–52]	NS
PM	3	12 [11–46]	NS
UM	2	43.5 [36–51]	0.011*

Significant differences in *p*-values are depicted by an * and bold text. NS values have a *p*-value >0.2.

Abbreviations P*-CYP2C19* phenoconverted phenotype CYP2C19; *P-CYP2D6*, phenoconverted phenotype CYP2D6; *NM*, normal metabolizer; *IM*, intermediate metabolizer; *PM*, poor metabolizer; *UM*, ultrarapid metabolizer; *ref* reference.

Patients with a non-psychotic main diagnosis had an odds ratio (OR) of 2.60 (95% CI: 1.04–6.54; adjusted OR 2.43; 95% CI: 0.90–6.55, Table 4) for higher UKU-score. Patients with polypharmacy had an OR of 4.47 (95% CI: 1.90–10.53; adjusted OR 4.26; 95% CI: 1.76–10.32) for higher UKU-score. No other statistical differences between UKU-score and covariates were found.

**TABLE 4:** Association between side effects with baseline characteristics and phenotypes of CYP-substrate users and UKU-score.

Total population	Number (%)	Total score on UKU categorized			OR (95% CI)	
		Low (≤11, %)	Moderate (12–20, %)	High (≥22, %)	Crude	Adjusted
Total with known UKU	104 (88.9)	37 (31.6)	34 (29.1)	33 (28.2)	N.A.	N.A.
*Age, categorized*					0.55 (0.25–1.25)	0.46 (0.18–1.19)
43 or younger (ref)	52 (50.0)	15 (28.8)	20 (38.5)	17 (32.7)		
44 or older	52 (50.0)	22 (42,3)	14 (26,9)	16 (30,8)		
*Gender*					1.34 (0.60–3.02)	1.25 (0.51–3.08)
Male (ref)	43 (41.3)	17 (39.5)	13 (30.2)	13 (30.2)		
Female	61 (58.7)	20 (32.8)	21 (34.4)	20 (32.8)		
*BMI, categorized*					1,44 (0.55–3.76)	1,42 (0.50–4.03)
< 30 (ref)	26 (25.2)	12 (46.2)	7 (26.9)	7 (26.9)		
30–40	51 (49.5)	19 (37.3)	21 (41.1)	11 (21.6)		
>40	26 (25.2)	6 (23.1)	6 (23.1)	14 (53.8)	2,86 (0.87–9.43)	2,64 (0.72–9.64)
*Main diagnosis*					2.60 (1,04–6.54)*	2,43 (0.90–6.55)
Psychotic (ref)	68 (65,4)	29 (42,6)	24 (35,3)	15 (22,1)		
Non-psychotic	36 (34,6)	8 (22,2)	10 (27,8)	18 (50,0)		
*Polypharmacy*					4.47 (1.90–10.53)*	4.26 (1.76–10.32)*
Yes	63 (60.5)	14 (22.2)	22 (34.9)	27 (42.9)		
No (ref)	41 (39.4)	23 (56.1)	12 (29.3)	6 (14.6)		
Only CYP-substrate users						
*P-CYP2C19^a^*	15 (14.4)				1.71 (0.13–22.51)	1.92 (0.09–49.40)
NM + UM (ref)	11	4 (36.4)	4 (36.4)	3 (27.3)		
IM + PM	4	1 (25)	1 (25)	2 (50)		
*P-CYP2D6^a^*	49 (47.1)				2.14 (0.62–7.39)	1.31 (0.30–5.68)
NM + UM (ref)	23	9 (39.1)	5 (21.7)	9 (39.1)		
IM + PM	26	6 (23.1)	9 (34.6)	11 (42.3)		
*CYP2C19^a^*	15 (14.4)				N.A.^b^	N.A.^b^
NM + UM (ref)	12	5 (41.7)	4 (33.3)	3 (25.0)		
IM + PM	3	0 (0)	1 (33.3)	2 (66.7)		
*CYP2D6^a^*	49 (47.1)				1.33 (0.39–4.58)	0.74 (0.17–3.30)
NM + UM (ref)	27	9 (33.3)	6 (22.2)	12 (44.4)		
IM + PM	22	6 (27.3)	8 (36.4)	8 (36.4)		

Significant differences in *p*-values are depicted by an * and bold text. NS values have a *p*-value >0.2.

Abbreviations *P-CYP2C19* phenoconverted phenotype CYP2C19; *P-CYP2D6* phenoconverted phenotype CYP2D6; *NM*, normal metabolizer; *IM*, intermediate metabolizer; *PM*, poor metabolizer; *UM*, ultrarapid metabolizer, ref reference, 95*% CI* 95% confidence interval.

^a^ The different phenotype groups were combined because separately the sample size of the phenotype groups was too small to perform logistic regression.

^b^ The number of patients with *CYP2C19* IM and PM is too small to perform logistic regression, therefore no result can be given for *CYP2C19*.

There was no increase of the total UKU-score associated with the genotype-predicted phenotypes of *CYP2C19* and *CYP2D6* (Table 3; Table 4). For *P-CYP2D6*, there was a significant difference between the total UKU-score of the NM (median 14) and UM (median 43.5, *p* < 0.05). There were no significant differences for IM (median 19.5) and PM (median 15). For *P-CYP2C19*, no increase in total UKU-score was seen considering the different phenotypes.

No significant differences were seen in the prevalence of certain side effects comparing the different genotype-predicted phenotypes of *CYP2C19* (Table 5) and *CYP2D6* (Table 6). Considering phenoconversion, several differences were noted. For *P-CYP2C19*, there was a higher prevalence of nausea and/or vomiting in the IM and PM group (75.0%) compared to the NM and UM group (9.1%, *p* < 0.05, Table 5). There were no further side effects with significant between-group differences. Comparing the prevalence of specific side effects and *P-CYP2D6*, there was a significant difference in the prevalence of depression (Table 6). 30.4% of the NMs and UMs experienced this side effect compared to 64.0% of the IMs and PMs. The IMs and PMs also experienced a higher rate of increased dream activity (48.0%) compared to the NMs and UMs (21.7%), but this result was not statistically significant (*p* = 0.057). The same is true for sleepiness, which 76.0% of the IMs and PMs experienced compared to 52.2% of the NMs and UMs (*p* = 0.085), and nausea and/or vomiting, which 36,0% of the IMs and PMs experienced compared to 13,0% for the NMs and UMs (*p* = 0.067). More patients in the NM and UM group of *P-CYP2D6* (13.6%) experienced gynecomastia, compared to 0% of the IMs and PMs. However, this was also not statistically significant (*p* = 0.095). Other side effects did also not show any significant differences.

**TABLE 5:** Comparison of specific CYP-related side-effects and phenotype of CYP2C19 in CYP2C19-substrate users. The side effects epileptic seizures, amenorrhea, galactorrhea and gynecomastia were not included because they were not present in any of the patients.

Item	Total % (*n* = 15)	CYP2C19^a^			P-CYP2C19^a^		
		NM + UM % (*n* = 12)	IM + PM % (*n* = 3)	*p*-value	NM + UM % (*n* = 11)	IM + PM % (*n* = 4)	*p*-value
*Fatigue*	60.0	58.3	66.7	NS	63.6	50.0	NS
*Sleepiness*	66.7	58.3	100	NS	54.5	100	NS
*Depression*	33.3	25.0	66.7	NS	27.3	50.0	NS
*Tension/Inner unrest*	46.7	41.7	66.7	NS	45.5	50.0	NS
*Increased duration of sleep*	20.0	16.7	33.3	NS	18.2	25.0	NS
*Reduced duration of sleep*	13.3	16.7	0	NS	9.1	25.0	NS
*Increased dream activity*	33.3	33.3	33.3	NS	36.4	25.0	NS
*Paresthesia*	6.7	0	33.3	0.200	0	25.0	NS
*Increased salivation*	26.7	33.3	0	NS	27.3	25.0	NS
*Dry mouth*	66.7	66.7	66.7	NS	72.7	50.0	NS
*Nausea/vomiting*	26.7	16.7	66.7	0.154	9.1	75.0	0.033*
*Diarrhea*	13.3	16.7	0	NS	18.2	0	NS
*Constipation*	26.7	25.0	33.3	NS	27.3	25.0	NS
*Micturition disturbances*	13.3	8.3	33.3	NS	9.1	25.0	NS
*Orthostatic dizziness*	46.7	50	33.3	NS	54.5	25.0	NS
*Palpitations/tachycardia*	46.7	41.7	66.7	NS	45.5	50.0	NS
*Increased tendency to sweating*	20.0	16.7	33.3	NS	18.2	25.0	NS
*Rash*	46.7	41.7	66.7	NS	45.5	50.0	NS
*Pruritus*	33.3	33.3	33.3	NS	36.4	25.0	NS
*Weight gain*	26.7	25.0	33.3	NS	27.3	25.0	NS
*Weight loss*	13.3	16.7	0	NS	18.2	0	NS
*Gynecomastia*	6.7	8.3	0	NS	9.1	0	NS
*Increased sexual desire*	20.0	16.7	33.3	NS	18.2	25.0	NS
*Diminished sexual desire*	20.0	25.0	0	NS	27.3	0	NS
*Erectile dysfunction*	13.3	8.3	33.3	NS	9.1	35.0	NS
*Ejaculatory dysfunction*	6.7	8.3	0	NS	9.1	0	NS
*Orgasmic dysfunction*	40.0	41.7	33.3	NS	45.5	25.0	NS
*Dry vagina*	6.7	0	33.3	0.200	0	25.0	NS
*Headache*	13.3	8.3	33.3	NS	9.1	25.0	NS

Significant differences in *p*-values are depicted by an * and bold text. NS, values have a *p*-value >0.2.

Abbreviations *P-CYP2C19* phenoconverted phenotype CYP2C19; *NM*, normal metabolizer; *IM*, intermediate metabolizer; *PM*, poor metabolizer; *UM*, ultrarapid metabolizer.

^a^ The different phenotype groups were combined because separately the sample size of the phenotype groups was too small.

**TABLE 6:** Comparison of specific CYP-related side-effects and phenotype of CYP2D6 in CYP2D6-substrate users. The side effects epileptic seizures, amenorrhea and galactorrhea were not included because they were not present in any of the patients.

Item	Total % (*n* = 48)	CYP2D6^a^			P-CYP2D6^a^		
		NM + UM % (*n* = 27)	IM + PM % (*n* = 21)	*p*-value	NM + UM % (*n* = 23)	IM + PM % (*n* = 25)	*p*-value
*Fatigue*	66.7	66.7	66.7	NS	65.2	68.0	NS
*Sleepiness*	64.6	55.6	76.2	0.138	52,2	76.0	0.085
*Depression*	47.9	40.7	57.1	NS	30.4	64.0	0.020*
*Tension/Inner unrest*	56.3	55.6	57.1	NS	47.8	64.0	NS
*Increased duration of sleep*	20.8	22.2	19.0	NS	21.7	20.0	NS
*Reduced duration of sleep*	22.9	25.9	19.0	NS	17.4	28.0	NS
*Increased dream activity*	35.4	33.3	38.1	NS	21.7	48.0	0.057
*Paresthesia*	18.8	25.9	9.5	0.149	26.1	12.0	NS
*Increased salivation*	22.9	22.2	23.8	NS	21.7	24.0	NS
*Dry mouth*	45.8	51.9	38.1	NS	47.8	44.0	NS
*Nausea/vomiting*	25.0	18.5	33.3	NS	13.0	36.0	0.067
*Diarrhea*	14.6	14.8	14.3	NS	17.4	12.0	NS
*Constipation*	16.7	11.1	23.8	NS	8.7	24.0	NS
*Micturition disturbances*	10.4	7.4	14.3	NS	8.7	12.0	NS
*Orthostatic dizziness*	43.8	48.1	38.1	NS	43.5	44.0	NS
*Palpitations/tachycardia*	41.7	37.0	47.6	NS	39.1	44.0	NS
*Increased tendency to sweating*	27.1	33.3	19.0	NS	34.8	20.0	NS
*Rash*	22.9	25.9	19.0	NS	30.4	16.0	NS
*Pruritus*	31.3	29.6	33.3	NS	34.8	28.0	NS
*Weight gain*	33.3	33.3	33.3	NS	34.8	32.0	NS
*Weight loss*	25.0	22.2	28.6	NS	17.4	32.0	NS
*Menorrhagia*	6.3	3.7	9.5	NS	4.3	8.0	NS
*Gynecomastia*	6.4	11.5	0	NS	13.6	0	0.095
*Increased sexual desire*	10.4	11.1	9.5	NS	13.0	8.0	NS
*Diminished sexual desire*	18.8	18.5	19.0	NS	21.7	16.0	NS
*Erectile dysfunction*	14.6	14.8	14.3	NS	17.4	12.0	NS
*Ejaculatory dysfunction*	6.3	3.7	9.5	NS	4.3	8.0	NS
*Orgasmic dysfunction*	22.9	18.5	28.6	NS	21.7	24.0	NS
*Dry vagina*	8.3	7.4	9.5	NS	8.7	8.0	NS
*Headache*	22.9	22.2	23.8	NS	17.4	28.0	NS

Significant differences in *p*-values are depicted by an * and bold text. NS, values have a *p*-value >0.2.

Abbreviations *P-CYP2D6*, phenoconverted phenotype CYP2D6; *NM*, normal metabolizer; *IM*, intermediate metabolizer; *PM*, poor metabolizer’; *UM*, ultrarapid metabolizer.

^a^ The different phenotype groups were combined because separately the sample size of the phenotype groups was too small.

**FIGURE 3:**
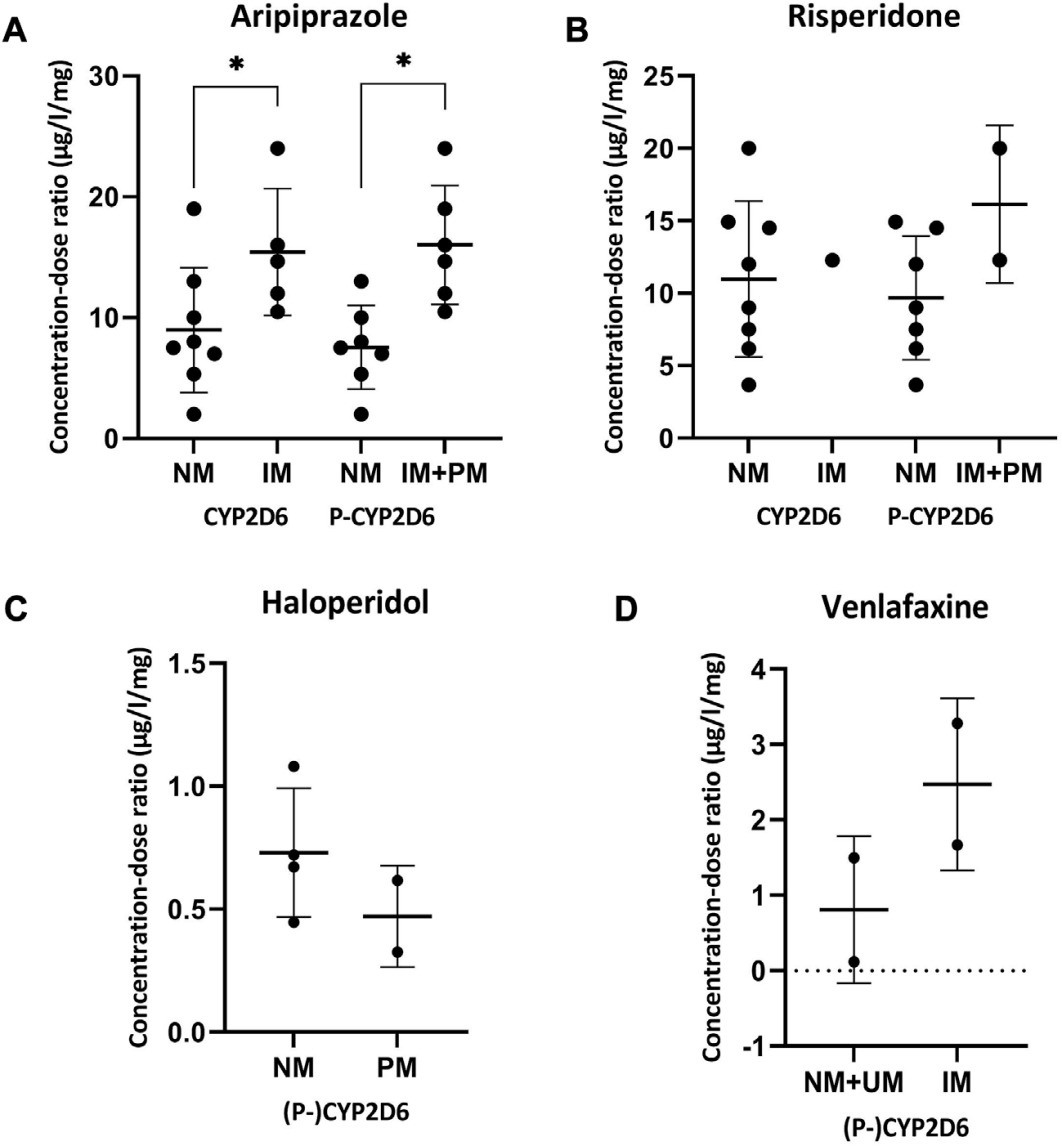
Concentration-dose ratios with SD of aripiprazole **(A)**, risperidone **(B)**, haloperidol **(C)** and venlafaxine **(D)** and the phenoconverted phenotype of CYP2D6. Significant differences (*p* < 0.05) are depicted with an *. The different phenotype groups were combined because separately the sample size of the phenotype groups was too small. For haloperidol and venlafaxine there was no difference in the patients in the different phenotype groups considering phenoconversion or not. Abbreviations *P-CYP2C19*, phenoconverted phenotype CYP2C19; *P-CYP2D6*, phenoconverted phenotype CYP2D6; *SD*, standard deviation; *NM*, normal metabolizer; *IM*, intermediate metabolizer; *PM*, poor metabolizer; *UM*, ultrarapid metabolizer.

For the comparison of the CD-ratio, the number of patients was sufficient for the analysis of CYP2D6-substrates aripiprazole, risperidone, haloperidol and venlafaxine. From the data in Figure 3, it was apparent that the concentration-dose ratio of aripiprazole is significantly higher for IMs and PMs than NMs with (7.55 ± 3.46 μg/l/mg versus 16.03 ± 4.92 μg/l/mg) and without (8.98 ± 5.16 μg/l/mg versus 15.43 ± 5.25 μg/l/mg) considering phenoconversion. For risperidone, haloperidol and venlafaxine no statistic significant differences were seen.

## 4 Discussion

This study shows that in 10% of the psychiatric patients any form of phenoconversion, where the predicted phenotype shifts based on genotype and co-medication, occurred. It also plays a role in the side effects experienced by these patients. This study shows that specific side effects such as nausea and depression are more prevalent in patients with an IM or PM phenoconverted phenotype of *CYP2C19* and *CYP2D6*.

In this study population, there were significantly more *CYP2C19* UM patients compared to the control population consisting of CAD-patients. In the Dutch Caribbean population, in which the phenotype distribution is comparable to Caucasians, a study found no differences at all in the prevalence of specific *CYP2D6* or *CYP2C19* phenotypes in psychiatric patients (Koopmans et al., 2017). This difference may be explained by the fact that the UMC Utrecht is a tertiary care center, to which patients are only referred if the treatment in the first or secondary line of care was not adequate. In a previous study in an American tertiary psychiatric hospital, a higher prevalence of genetic variants leading to a phenotype other than NM was seen (Ruaño et al., 2008). Looking at the distribution of phenotypes before and after phenoconversion, there were specifically more *P-CYP2D6* PM patients. These findings seem to be consistent with the existing literature (Preskorn et al., 2013; Mostafa et al., 2019). This shows it is important to consider phenoconversion when predicting a patient’s phenotype. For *CYP2C19* and *CYP2D6*, the phenotype influences the efficacy and tolerability of antidepressants and consequently there are different pharmacotherapeutic recommendations for each specific genotype-predicted phenotype (Gressier et al., 2015; Brouwer et al., 2022; Campos et al., 2022). There are also pharmacotherapeutic recommendations available for antipsychotics (Beunk et al., 2023). So, if the genotype-predicted phenotype shifts because of phenoconversion, it is possible that other recommendations are given.

Based on the total UKU-score, no statistically significant associations were found between the genotype-predicted or phenoconverted phenotype and amount and/or severity of side effects. However, the OR of the *CYP2D6* phenoconverted phenotype was 1.31 and higher than 0.74, the OR of the *CYP2D6* genotype-predicted phenotype. No comparison could be made for *CYP2C19* because no logistic regression could be performed in the genotype-predicted group. In other studies, it has also been seen that there is a stronger association between phenoconverted phenotype and antidepressant efficacy and not necessarily between genotype-predicted phenotype and the antidepressant efficacy (Gressier et al., 2015).

This study found that there are also some non-genetic factors that influence the UKU-score of psychiatric patients. Patients who have polypharmacy have an OR of 4.26 for a moderate or high total UKU-score and therefore experience more or more severe side effects. These results are consistent with other studies, the higher the number of drugs a patient uses, the higher the chance of side effects and drug-drug interactions (Maher et al., 2014; Malki and Pearson, 2020).

A non-psychotic diagnosis also seemed to have an influence on the amount of side effects experienced. LL-patients with a non-psychotic diagnosis had a crude OR of 2.60 compared to LL-patients with a psychotic diagnosis. However, this result was not statistically significant when it was corrected for other covariables. The relationship between diagnosis and side effects is not clear, because several other underlying factors can play a role. The diagnosis of patients not only tells us something about the psychiatric disease, but also about the possible pharmacotherapy with associated side effects. Moreover, patients enrolled in the LL-clinic typically have more complex and more persistent mental disorders and therefore the current pharmacotherapy may lead to more side effects.

BMI may also play a role in the amount and/or severity of the side effects experienced. Patients with a BMI > 40 had a median UKU-score of 23.5, which was significantly higher than patients with a BMI between 30 and 40 or lower than 30, who respectively had scores of 15 and 13. However, this result was not seen when looking at the association between the UKU-score and the BMI as the OR was not statistically significant. Patients with a higher BMI often have a different response to antidepressants or antipsychotics, most of the time needing higher doses and thus experiencing more side effects (Warrings et al., 2021). Obesity and psychiatric disease have a complex bidirectional relationship (Holt and Peveler, 2009; Woo et al., 2016). Therefore, it is important to also consider the weight effects of a certain drug when choosing effective treatment for the mental disorder (McElroy, 2009). These aspects are already incorporated into the LL-clinic at the UMC Utrecht (UMC Utrecht, 2022a).

In this study it was also possible to look at the prevalence of CYP-specific side effects. It was found that 75.0% of the *P-CYP2C19* IMs and PMs experience nausea and vomiting compared to 9.1% of the NMs and UMs. Another study also found *CYP2C19* PMs have a higher chance of gastro-intestinal side effects when using an SSRI (Fabbri et al., 2018). Side effects that occurred more often in *P-CYP2D6* IMs and PMs were depression, increased dream activity and sleepiness. 64.0% *P-CYP2D6* IMs and PMs experienced the side effect depression compared to 30.4% of the NMs and UMs. The side effect depression is associated with antipsychotic use, possibly related to hyperprolactinemia (Milano et al., 2017). However, the relationship between hyperprolactinemia and *CYP2D6* phenotype is unclear (Calafato et al., 2020). For the sleep-related side effects, some research indicates that these side effects are CYP-related, but different factors may influence this. For example, patients using a SSRI metabolized by CYP2D6 more often report nightmares as a side effect (Eugene, 2019). However, it also seems that psychiatric patients in general more often have more vivid dreams (Schredl and Schredl, 2018). The same goes for the relationship between sleep and psychiatric disorders (Krystal, 2012). Gynecomastia was a side effect more often seen in *P-CYP2D6* NM + UM group. However, this side effect occurred specifically by the two UM patients. These two patients had a very complex background, having multiple comorbidities and/or switching drugs when the questionnaire was filled out, and therefore it is unsure if the occurrence of gynecomastia is due to the phenotype of these patients.

Lastly, next to the UKU-results, there was also an analysis of the drug concentration in plasma, corrected for the dose and phenotype. A two times higher CD-ratio was seen for (*P-*)*CYP2D6* IMs and PMs, which was statistically significant. These results are consistent with the findings of previous work (Jukic et al., 2019; Kiss et al., 2020).

This study has several strengths. First, general genotyping (i.e., without a specific reason) is not standard clinical practice for psychiatric patients. However, for each patient enrolled in the LL-clinic, a psychiatric PGx panel was performed. Moreover, this study considered phenoconversion, specifically by co-medication, and its effect on the phenotype. Most studies in the field currently focus on the genotype-predicted phenotype and do not take the effect of co-medication into account (Shah et al., 2016). Specifically in the psychiatric population there is a lot of drugs that are known CYP-modulators, so it is an important factor (Flockhart et al., 2022). For future research it is important to look at other factors that influence phenoconversion such as smoking, alcohol consumption and disease state (Klomp et al., 2020). Third, the UKU is a validated standardized questionnaire, specifically for psychiatric drug-users (Lingjærde et al., 1987).

The current study was, however, limited by the sample size. Only one patient per week was enrolled in the LL-clinic. This could be a limitation specifically for phenotypes like UM and PM because of their lower prevalence. Moreover, not every patient used a CYP-substrate drug and therefore not all patients could be included in the analysis of CYP-substrate users and phenotype and phenotype groups had to be combined for some of the analyses. To include enough PMs and UMs, large-sample prospective trials need to be conducted. Another limitation is the data collection. Genotypes and drug use had to be obtained manually. To minimize the chance of errors, this was done in a standardized manner. Lastly, extrapyramidal symptoms were not included in the adapted version of the UKU-questionnaire. Also, although side effects were collected through the validated UKU-questionnaire, some side effects may need to be objectified using laboratory tests or physical examinations (Lingjærde et al., 1987).

In conclusion, this study shows that phenoconversion is important to consider when looking at a patient’s genotype. In the psychiatric population, where a difference in genotype distribution is observed, this phenomenon causes a shift from one phenotype to another. Although no significant associations were found between the phenotype and side effects experienced, there was a difference in the occurrence of specific side effects for the different phenoconverted phenotypes. More research on this topic is important to take the next step towards better prediction of a patient’s phenotype and possible prevention of side effects, contributing to personalized medicine.

## Data Availability

The original contributions presented in the study are included in the article/Supplementary Material, further inquiries can be directed to the corresponding author.

## Abbreviations

CAD, Coronary artery disease; DGI, Drug-gene interaction; DPWG, Dutch Pharmacogenetics Working Group; EPS, Extrapyramidal symptoms; LL-clinic, “Body and Life” outpatient clinic (Dutch: *Lijf en Leven*); LL-patients, Patients from “Body and Life” outpatient clinic; P-CYP2C19, Phenotype CYP2C19 after phenoconversion; P-CYP2D6, Phenotype CYP2D6 after phenoconversion; UKU, *Udvalg for Kliniske Undersølger* side effects rating scale.
