# Supplementary material for: Pharmacogenetics and phenoconversion: the influence on side effects experienced by psychiatric patients
Source: Front Genet. 2023 Aug 25;14:1249164. doi: 10.3389/fgene.2023.1249164 (PMC10486269; doi:10.3389/fgene.2023.1249164)
Supplement: Supplementary file 1 [file Table1.DOCX]

Supplementary Material

Pharmacogenetics and phenoconversion: the influence on side effects experienced by psychiatric patients

Manon G. den Uil^1^, Lotte H.W. Hut^1^, Kay R. Wagelaar^1,2^, Heshu Abdullah-Koolmees^3,4^, Wiepke Cahn^5^, Ingeborg Wilting^1^, Vera H.M. Deneer^*^

*** Correspondence:** Vera H.M. Deneer
[v.h.m.deneer@umcutrecht.nl](mailto:v.h.m.deneer@umcutrecht.nl)

# Supplementary material 1

***Table S1.*** *Relevant inhibitors and inducers for CYP2C19 and CYP2D6 phenoconversion.* The division is based on the Flockhart Table.

| CYP2C19 | CYP2D6 |
| --- | --- |
| *Strong inhibitor*  Fluvoxamine | *Strong inhibitor*  Bupropion  Fluoxetine  Quinidine  Paroxetine |
| *Moderate inhibitor*  Esomeprazole  Fluoxetine | *Moderate inhibitor*  Doxepin  Duloxetine  Moclobemide |
| *Inducer*  Carbamazepine  St. John’s wort |  |

*References*

Flockhart DA, Thacker D, McDonald C DZ. The Flockhart Cytochrome P450 Drug-Drug Interaction Table. Division of Clinical Pharmacology, Indiana University School of Medicine (Updated 2021). [Internet]. [cited 2022 Mar 8]. Available from: <https://drug-interactions.medicine.iu.edu/>

# Supplementary material 2

Side effects were evaluated using the the *Udvalg for Kliniske Undersølgelser side effects rating scale (UKU)* for the registration of side effects of psychotropic drugs. (26) The UKU is a four-point scale, and all scores were added together to get a total score. A higher score implies a higher rate of side effects or more severe side effects. An adapted version of the UKU-rating scale specifically for the LL-clinic was used, from which the extrapyramidal symptoms were excluded from the category neurologic side effects. An overview of the side effects which were evaluated, can be found below. The full version of the UKU with the correct formulation of the questions can be found here (<https://scnp.org/fileadmin/SCNP/SCNP/UKU/UKU-Pat-%20English%20.pdf>). In the outpatient clinic, a translated version of the UKU is used. The UKU-questionnaire was conducted orally by a nurse specialist via a semi structured interview with the patient.

*Side effects evaluated*

1. Psychic
   1. Concentration Difficulties
   2. Asthenia/Lassitude/Increased Fatigability
   3. Sleepiness/Sedation
   4. Failing Memory
   5. Depression
   6. Tension/Inner Unrest
   7. Increased Duration of Sleep
   8. Reduced Duration of Sleep
   9. Increased Dream Activity
   10. Emotional indifference
2. Neurologic
   1. Epileptic Seizures
   2. Parasthesias
3. Autonomic
   1. Accommodation Disturbances
   2. Increased Salivation
   3. Reduced Salivation
   4. Nausea/Vomiting
   5. Diarrhoea
   6. Constipation
   7. Micturition Disturbances
   8. Polyuria/Polydipsia
   9. Orthostatic Dizziness
   10. Palpitations/Tachycardia
   11. Increased Tendency to Sweating
4. Other
   1. Rash

- 4.1.a – Morbilliform
- 4.1.b – Petechial
- 4.1.c – Urticarial
- 4.1.d – Psoriatic
- 4.1.e – Cannot be classified
  1. Pruritis
  2. Photosensitivity
  3. Increased Pigmentation
  4. Weight gain
  5. Weight Loss
  6. Menorrhagia
  7. Amenorrhoea
  8. Galactorrhoea
  9. Gynaecomastia
  10. Increased Sexual Desire
  11. Diminished Sexual Desire
  12. Erectile Dysfunction
  13. Ejaculatory Dysfunction
  14. Orgastic Dysfunction
  15. Dry Vagina
  16. Headache
  17. Physical Dependence
  18. Psychic Dependence

.
